# The risk factors of thrombus formation and the effect of catheter ablation on repetitive thrombus formation in patients with atrial fibrillation: a single center retrospective study in China

**DOI:** 10.1186/s12872-023-03050-z

**Published:** 2023-01-17

**Authors:** Huiyu Liu, Mingjie Lin, Wenqiang Han, Junye Ge, Kellina Maduray, Jingquan Zhong

**Affiliations:** 1 The Key Laboratory of Cardiovascular Remodeling and Function Research, Chinese Ministry of Education, Chinese National Health Commission and Chinese Academy of Medical Sciences, The State and Shandong Province Joint Key Laboratory of Translational Cardiovascular Medicine, Department of Cardiology, Qilu Hospital of Shandong University, Jinan, China; 2 Department of Cardiology, Qilu Hospital (Qingdao) of Shandong University, Qilu Hospital (Qingdao), Cheeloo College of Medicine, Shandong University, 758 Hefei Road, Qingdao, 266035 Shandong China; 3 Department of Cardiology, Nanyang Central Hospital, Nanyang, China

**Keywords:** Atrial fibrillation, Catheter ablation, Left atrial appendage, Thrombus formation

## Abstract

**Background:**

Atrial fibrillation (AF) predisposes patients to the formation of atrial thrombi. The CHA_2_DS_2_-VASc score does not include all risk factors for atrial thrombosis. The present study is designed to explore the influencing factors of thrombus formation in patients with AF and to investigate the effect of catheter ablation (CA) on recurrent thrombosis in patients with a history of intracardiac thrombus.

**Methods:**

(1) This study consisted of 1726 patients that underwent CA, among which 58 patients had a history of intracardiac thrombus prior to CA. The risk factors for thrombus formation were explored by comparing the baseline clinical characteristics of patients with and without atrial thrombus. (2) The left atrial appendage flow velocity (LAAFV) in patients with a history of intracardiac thrombus who were willing to undergo transesophageal echocardiography (TEE) at the latest follow-up were examined, and comparisons of the LAAFV was made before and after CA.

**Results:**

The median follow-up period is 13 months. Persistent AF was found to be the only independent risk factor affecting the formation of atrial thrombus among the investigated factors (OR 3.152; 95%CI 1.806–5.500; *p* < 0.001). Twenty-seven patients agreed to undergo TEE during follow-up, no clinical ischemic stroke events were recorded, no recurrent intracardiac thrombus formation was detected in patients, 15 patients maintained sinus rhythm (55.6%) during follow-up; successful CA significantly increased LAAFV (difference between latest evaluation prior to CA 17.46 ± 14.81 cm/s, *p* < 0.001).

**Conclusions:**

Persistent AF is the only independent risk factor for thrombus formation. Successful CA may improve the LAAFV and thereby decrease the risk of intracardiac thrombus formation.

**Supplementary Information:**

The online version contains supplementary material available at 10.1186/s12872-023-03050-z.

## Introduction

Atrial fibrillation (AF) is the most common sustained cardiac arrhythmia in adults [1]. AF predisposes patients to the formation of atrial thrombi (about 10%), the source of thromboembolic events [2]. The CHA_2_DS_2_-VASc score (including congestive heart failure, hypertension, age ≥ 75 years, diabetes mellitus, stroke, vascular disease, age 65–74 years, sex category) is the most predominantly used AF stroke risk stratification scheme to predict thromboembolic events [3]. However, even patients with low risk stratification were found thromboembolic events. The CHA_2_DS_2_-VASc score does not include all risk factors for intra-cardiac thrombosis [4, 5] and AF patients cannot be evaluated comprehensively according to the CHA_2_DS_2_-VASc score.

Transesophageal echocardiography (TEE) is the most reliable examination to assess the presence of atrial thrombosis. The current clinical practice guidelines recommend using vitamin K antagonists (VKA) [6] or direct-acting oral anticoagulants (DOAC) for the treatment of intracardiac thrombus [7]. After resolving the clot, further treatment measures, such as continuing anticoagulants or antiarrhythmics, are determined by the treating physician. Effective measures may prevent patients from recurrent thrombus formation; moreover, it will minimize the possibility of a secondary cardioembolic stroke. Catheter ablation (CA) is an effective modality to restore sinus rhythm in AF [8] and to increase left atrial appendage flow velocity (LAAFV), thereby reducing and even reversing the risk of thrombus formation [9, 10]. Nevertheless, the effect of CA on thrombus formation in AF patients with intracardiac thrombus history has not yet been explored. This study can be divided into two parts: (1) Assessment of intracardiac thrombus risk factors in patients with nonvalvular atrial fibrillation (NVAF). (2) Evaluation of sinus rhythm maintenance, repetitive thrombus formation, and ischemic brain lesions after CA in AF patients with a previously resolved thrombus with a minimum follow-up of one year.

## Methods

### Study population

**Fig. 1:**
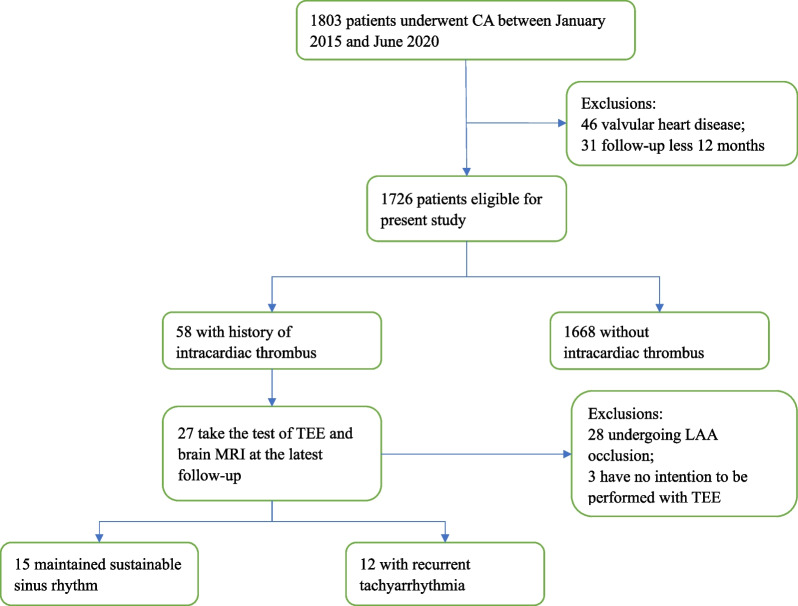
Screening process

The present study was based on data obtained from a prospective observational study (Chinese Clinical Trial Registry: ChiCTR-OCH-14004674). The study was approved by the Institutional Ethics Committee; all participants signed informed consent. This single-center study reviewed 1803 patients with AF undergoing CA between January 2015 and June 2020. Seventy-seven patients were excluded due to mitral stenosis, severe mitral regurgitation, valve prosthesis, or a follow-up failing to exceed 12 months. Therefore, 1726 patients were included to analyze the risk factor of thrombus formation, among which 58 patients had a history of intracardiac thrombus diagnosed with routine TEE prior to CA. Twenty-seven patients underwent TEE and brain magnetic resonance imaging (MRI) at the latest follow-up (Fig. 1).

### Data collection

Data were obtained from the medical record information of Qilu Hospital, including demographic data, previous disease history, transthoracic echocardiographic examinations, and laboratory results. AF type, coronary heart disease, hyperlipidemia, congestive heart failure, hypertension, diabetes mellitus, stroke/transient ischemic attacks were determined according to relevant guidelines or expert consensus. Liver dysfunction was defined as elevated alanine aminotransferase, aspartate aminotransferase, and gamma-glutamyl transferase. Renal dysfunction was defined as eGFR < 60 mL/min/1.73 m^2^. Anticoagulation was defined as a history of anticoagulant therapy before CA, and it was detailed to warfarin and DOAC categories. CHA_2_DS_2_-VASc score was calculated as per the guidelines [11]. According to the examination results, the patients were divided into non-intracardiac thrombus and intracardiac thrombus groups to analyze associated risk factors. The intracardiac thrombus group included patients with cardiac thrombi formed before CA.

As for the 58 patients with a history of intracardiac thrombus, 28 were excluded since they had undergone LAA occlusion, while 3 have no intention to be performed with TEE. So 27 patients agreed to be examined by TEE and brain MRI at the latest follow-up (June 2021), they were devided into sustainable sinus rhythm group and recurrent tachyarrhythmia group (Fig. 1).

### Transesophageal echocardiography examination and thrombus management

According to previous reports, the proportion of patients undergoing anticoagulation prior to CA is relatively low in China [12]. Therefore the 3 week pre-ablation anticoagulation was omitted in this study. In our center, TEE was routinely performed within 24 h prior to CA to assess for the presence of intracardiac thrombus. TEE was conducted by an experienced physician and confirmed by a senior cardiologist [13]. Spontaneous echo contrast (SEC) was ascertained by the presence of dynamic swirling smoke-like echos within the left atrium (LA) and the LAA using standard gain settings. LA thrombus was defined as an echodense intracavitary mass distinct from the underlying endocardium, not caused by pectinate muscles [14]. In addition to detecting intracardiac SEC and thrombus formation, LAAFV was also measured and recorded during the emptying phases. If a thrombus was detected, patients were administered with warfarin or DOACs under physician–patient joint decision. Heparin bridging is used for patients who therapeutically anticoagulated with warfarin. After a minimum of 8 weeks, a second TEE was conducted. If the thrombus failed to resolve, DOACs were replaced with warfarin and vice versa. The patient was then reassessed after 3 months, if resolved, CA was performed.

### Electrophysiological mapping and catheter ablation

The procedure of radiofrequency CA was detailed briefly as following. 3D electro-anatomical mapping (CARTO 3, Johnson and Johnson, Inc.) using a circular pulmonary vein mapping catheter (Lasso, Biosense-Webster Inc.) was conducted. The ablation was carried out with an open-irrigation 3.5 mm-tip deflectable catheter (SmartTouch Johnson and Johnson, Inc, 25–30 W, 43 °C). All patients underwent circumferential pulmonary vein isolation in their first procedure. In patients with persistent AF, complex fractionated atrial electrograms were targeted or additional lesion lines (mitral isthmus line, roof line) were made. The ablation endpoint was bidirectional block. If patients experienced cavotricuspid isthmus-dependent atrial flutter documented by electrocardiogram, cavotricuspid isthmus line ablation was performed. Non-Pulmonary vein foci or the superior vena cava was additionally ablated, under the operators’ discretion. Electrical cardioversion was performed if sinus rhythm was not achieved.

### Post-ablation management and follow-up

After CA, patients routinely took antiarrhythmic drugs, namely oral propafenone 0.45 g/day or amiodarone 0.2 g/day, and oral anticoagulants during the observational period (3 months); continuous anticoagulants was recommended for patients with a history of embolic events or recurrence of atrial tachyarrhythmia [15]. Antiarrhythmic drugs were managed according to the patients’ conditions and their individual decision. Post-ablation follow-up was conducted at the outpatient clinic at the 1st, 3rd, and 6th month, and every 6 months subsequently. Follow-up included 24 h-Holter monitoring and telephonic consultation every 3 months. If the patient presented with chest discomfort, a 12-lead electrocardiogram was conducted immediately. Recurrence was defined as atrial tachyarrhythmia lasting at least 30 s after the recommended observational period. The median follow-up period is 13 months (interquartile range 12, 15 months).

### Brain magnetic resonance imaging

Brain MRI was performed during the latest follow-up with a 1.5-T Siemens Magnetom scanner to identify ischemic and hemorrhagic cerebral lesions. The imaging protocol for all images consisted of a T2-weighted axial fluid-attenuated inversion recovery (FLAIR) sequence and a diffusion-weighted imaging (DWI) sequence. The parameters of the FLAIR sequence were as follows: TR 6000 ms, TE 140 ms, TI 2000 ms, slice thickness 6 mm, field of view 230 mm, and matrix 159 × 308. The parameters of the DWI sequence were as follows: TR 3000 ms, TE 99.3 ms, slice thickness 6 mm, field of view 230 mm, matrix 106 × 152, bandwidth 1538 Hz, and acquisition time of 26 s. For each DWI sequence, the apparent diffusion coefficient map was obtained to rule out a shine-through artifact. Embolic lesions, white matter hyperintensity, and microbleeds were defined according to the latest neuroimaging experts’ recommendations [16]. All MRI images were analyzed independently by a certified radiologist and certified physicians blinded to this study, clinical status, and identification of the patients.

### Statistical analysis

A chi-square analysis of the two groups was first performed (Table 1). Several variables (Cancer, Vascular disease, Hemorrhage and Anticoagulation) had a positive rate of 0 in the thrombus group, therefore were only present at baseline (Table 1) and could not be included in subsequent regression analyses (Table 2). The risk factors were evaluated with univariate and multivariable logistic analysis; variables with *p* ≤ 0.10 in the univariate analysis were included in the multivariable logistic regression model. This model accounted for clinically relevant baseline characteristics, including age, sex, body mass index (BMI), and the duration of AF. The clinical outcome is expressed as the hazard ratio (HR) and 95% confidence interval, which were calculated with the use of Cox regression. Missing values for continuous outcomes were imputed using the last observation carried forward to calculate the change from baseline over time, with post hoc confirmation achieved by means of multiple imputations.

**Table1:** Baseline clinical characteristics of the patients studied

Characteristics	Patients with atrial thrombus (n = 58)	Patients without atrial thrombus (n = 1668)	*p* value
Male, n (%)	39 (67.2)	1056 (63.3)	0.541
Age, years ± SD	61.5 ± 9.7	59.9 ± 10.5	
BMI, kg/m^2^			0.044*
≥ 24, n (%)	50 (86.2)*	1243 (74.5)*	
< 24, n (%)	8 (13.8)*	425 (25.5)*	
Time to AF diagnosis			0.130
≥ 2 years, n (%)	37 (63.8)	896 (53.7)	
< 2 years, n (%)	21 (36.2)	772 (46.3)	
AF type			< 0.001*
Paroxysmal AF, n (%)	20 (34.5)*	1056 (63.3)*	
Persistent AF, n (%)	38 (65.5)*	612 (36.7)*	
Cancer, n (%)	0 (0)	11 (0.7)	0.940
Coronary heart disease, n (%)	8 (13.8)	328 (19.7)	0.267
Hyperlipidemia, n (%)	6 (10.3)	257 (15.4)	0.315
LAD, mm			0.203
≥ 40, n (%)	34 (58.6)	836 (50.1)	
< 40, n (%)	24 (41.4)	832 (49.9)	
CHA_2_DS_2_-VASc score†			0.485
Low risk, n (%)	34 (58.6)	1053 (63.1)	
High risk, n (%)	24 (41.4)	615 (36.9)	
Congestive heart failure, n (%)	5 (8.6)	82 (4.9)	0.336
Hypertension, n (%)	27 (46.6)	769 (46.1)	0.946
Diabetes mellitus, n (%)	13 (22.4)	244 (14.6)	0.102
Stroke/TIA, n (%)	3 (5.2)	138 (8.3)	0.546
Vascular disease, n (%)	0 (0)	5 (0.3)	0.594
Hemorrhage, n (%)	0 (0)	9 (0.5)	0.845
Liver dysfunction, n (%)	1 (1.7)	122 (7.3)	0.172
Renal dysfunction, n (%)	17 (29.3)*	293 (17.6)*	0.022*
Anticoagulation, n (%)	0 (0)*	151 (9.0)*	0.046*
Warfarin	0 (0)	54 (3.2)	
DOAC	0 (0)	97 (5.8)	
Smoking, n (%)	22 (37.9)	518 (31.1)	0.267
Drinking, n (%)	18 (31.0)	477 (28.6)	0.687

*BMI* body mass index, *LAD* left atrial diameter, *TIA* transient ischemic attack

*p value < 0.05

^†^Low risk is defined as < 1 point in male and < 2 points in female patients. High risk is defined as ≥ 1 point in male or ≥ 2 points in female patients

**Table 2:** Univariate analysis and multivariable analysis of thrombus formation risk in patients with AF

Variables	Univariate regression OR (95% CI)	*p* Value	Multivariable regression OR (95% CI)	*p* value
Male	1.190 (0.681–2.077)	0.541	0.916 (0.502–1.670)	0.775
Age	1.015 (0.989–1.042)	0.253	1.021 (0.993–1.048)	0.139
BMI ≥ 24 kg/m^2^	2.137 (1.005–4.544)	0.049*	2.051 (0.952–4.420)	0.067
Time to AF diagnosis				
≥ 2 years	1.518 (0.881–2.616)	0.133	–	–
Persistent AF	3.278 (1.890–5.685)	< 0.001*	3.152 (1.806–5.500)	< 0.001*
Coronary heart disease	0.654 (0.307–1.392)	0.270	–	–
Hyperlipidemia	0.633 (0.269–1.490)	0.296	–	–
LAD ≥ 40 mm	1.410 (0.829–2.398)	0.205	–	–
High risk in CHA_2_DS_2_-VASc score†	1.209 (0.710–2.057)	0.485		
Congestive heart failure	1.825 (0.710–4.687)	0.212	–	–
Hypertension	1.018 (0.602–1.721)	0.946	–	–
Diabetes mellitus	1.686 (0.896–3.172)	0.105	–	–
Stroke/TIA	0.605 (0.187–1.958)	0.402	–	–
Liver dysfunction	0.222 (0.031–1.619)	0.138	–	–
Renal dysfunction	1.946 (1.090–3.473)	0.024*	1.778 (0.969–3.262)	0.063
Smoking	1.357 (0.790–2.329)	0.269	–	–
Drinking	1.124 (0.638–1.980)	0.687	–	–

*CI* confidence interval, *OR* odds ratio, *BMI* body mass index, *LAD* left atrial diameter, *TIA* transient ischemic attack

*p value < 0.05. †High risk is defined as ≥ 1 point in male or ≥ 2 points in female patients

Gaussian distribution was checked for the LAAFV and left atrial dimension (LAD) in patients with recurrence and no recurrence using the Kolmogorov–Smirnov test. The changes of LAAFV and LAD were compared by the Student’s *t*-test between prior CA and post-CA in non-recurrent and recurrent AF, respectively. The difference of LAAFV was compared by the Student’s *t*-test in sinus rhythm and AF rhythm prior and post ablation. All data analyses were operated by SPSS version 25.0 (IBM Corporation, Armonk, NY). The significance level was set at *p* < 0.05.

## Results

In this study, 58 out of the 1726 patients (3.36%) developed intracardiac thrombus. Baseline clinical and demographic characteristics of all patients are shown in Table 1. Regarding risk of cardiac thrombus formation, BMI, AF type, and renal dysfunction showed statistical significance (*p* < 0.10) between the two groups in the univariate analysis, which was included in the multivariate logistic model. The results demonstrated that persistent AF is the only independent risk factor affecting the formation of atrial thrombus (OR 3.152; 95%CI 1.806–5.500; *p* < 0.001, Table 2). The ablation procedure and periprocedural adverse events are shown in Table 3. During follow-up, the incidence of atrial tachyarrhythmia recurrence in the thrombus group was significantly higher than that of the no-thrombus group (23/58, 39.66% vs. 410/1668, 24.58%, *p* < 0.05). In the Cox regression analysis, after adjusting for age, sex, BMI, and the type of AF, patients with atrial thrombus had worse outcomes compared with those without atrial thrombus (HR 1.656; 95% CI 1.084–2.530; *p* = 0.020). The embolic events and death between the two groups had no statistical significance (Table 4).

**Table 3:** Ablation procedure and Periprocedural complications of the patients studied

Characteristics	Patients with atrial thrombus (n = 58)	Patients without atrial thrombus (n = 1668)
Detailed ablation procedure		
CPVI, n (%)	19 (32.8)	652 (39.1)
CPVI + CAFE, n (%)	11 (19.0)	297 (17.8)
CPVI + Linear, n (%)	11 (19.0)	358 (21.5)
CPVI + CAFE + Linear, n (%)	15 (25.9)	327 (19.6)
Others, n (%)	2 (3.4)	34 (2.0)
Periprocedural complications		
Cardiac tamponade, n (%)	0 (0)	2 (0.1)
Atrioesophageal fistula, n (%)	0 (0)	2 (0.1)
Major bleeding, n (%)	0 (0)	1 (0.1)
Stroke, n (%)	0 (0)	1 (0.1)
Hematoma at access site, n (%)	1 (1.72)	9 (0.5)

*CPVI* circumferential pulmonary vein isolation, *CFAE* complex fractionated atrial electrogram, *Linear* additional linear ablation

**Table 4:** Clinical outcome of the patients studied

End point	Patients with atrial thrombus (n = 58)	Patients without atrial thrombus (n = 1668)	Cox regression analysis of clinical outcome (95% CI)	*p* value
Recurrence, n (%)	23 (39.66)	410 (24.58)	1.656 (1.084–2.530)	0.020*
Embolism events, n (%)	1 (1.72)	20 (1.20)	1.345 (0.176–10.292)	0.775
Death, n (%)	0 (0)	17 (1.02)	0.000	0.983

^*^p value < 0.05

**Fig. 2:**
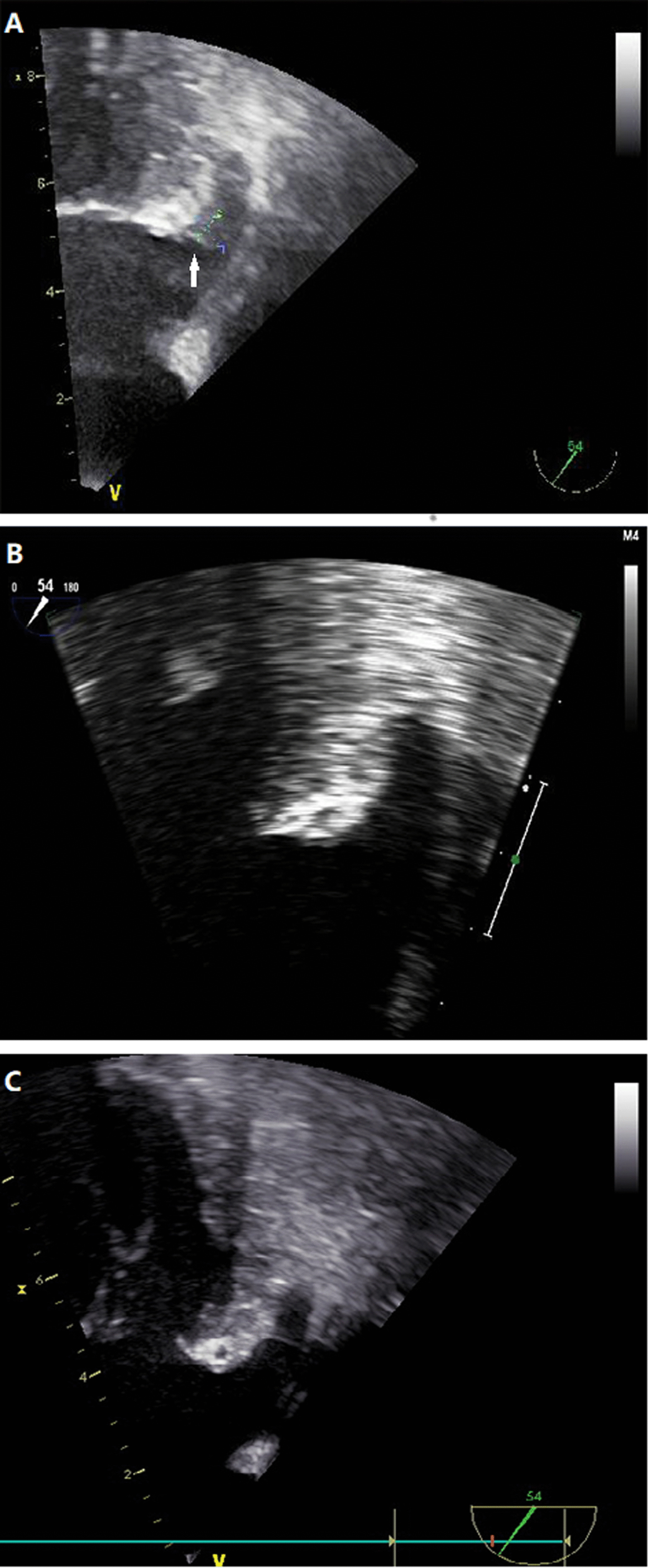
Typical LAA images for one patient with thrombus (white arrow) (**A**), post-resolution (**B**) and latest examination (**C**)

**Fig. 3:**
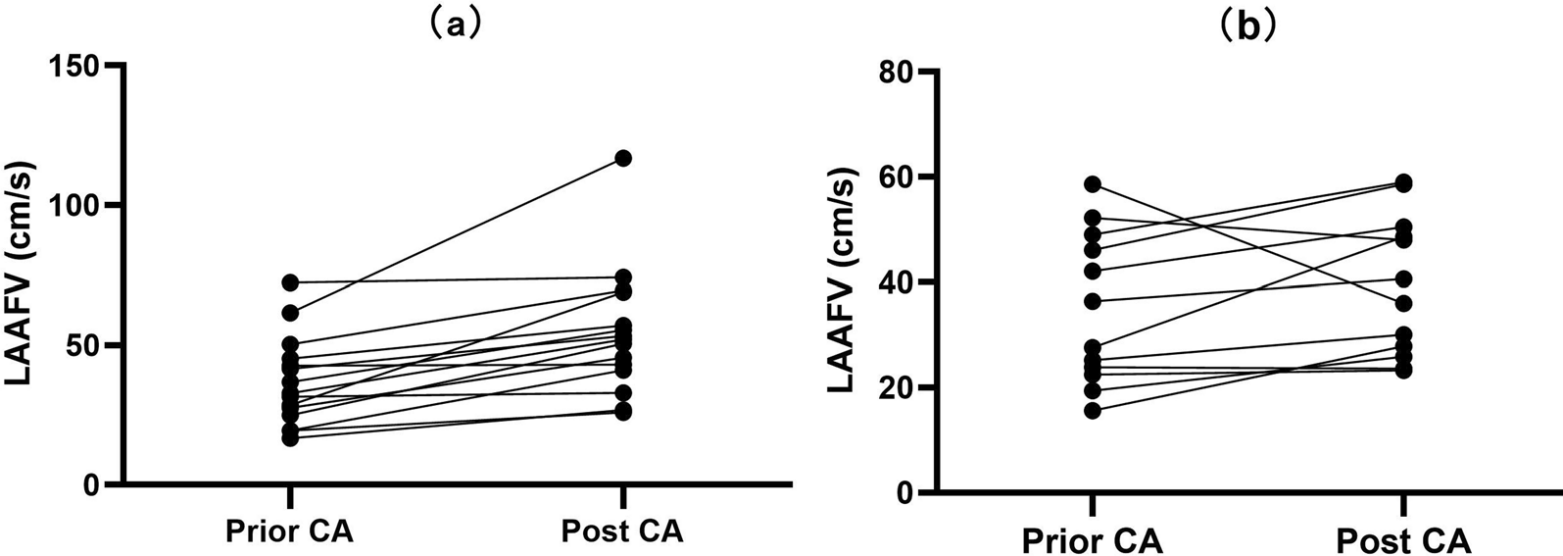
LAA flow velocity significantly increased among patients with sustainable sinus rhythm (**a**). No significant change was observed in recurrent AF patients (**b**)

Complete characteristics of the 27 patients with atrial thrombus are listed in Additional file 1: Table S1. For one patient (39 years old, persistent AF), typical LAA images with thrombus, post-resolution, and the latest examination are presented in Fig. 2. Of the 27 patients, thrombi were primarily located in the LAA (23/27, 85.19%); the CHA_2_DS_2_-VASc score was less than 1 for males and 2 for females in 16/27 (59.26%); 25 were treated with one type of anticoagulant (14/25 with dabigatran, 11/25 with warfarin) and 2 with dabigatran, following failure to resolve the thrombus with warfarin. Thrombus was resolved within 67 to 365 days; no major bleeding or clinical embolic events had been recorded. Fifteen patients attained sustainable sinus rhythm during 14 to 78 months follow-up since the last CA (Additional file 1: Table S2). To assess intracardial thrombus formation, LAAFV, and silent stroke, the 27 patients agreed to a post-CA TEE and brain MRI during the recent follow-up (Table 5 and Additional file 1: Table S3). Brain MRI findings included white matter hyperintensity (20/27), softening lesions (5/27), and no abnormalities (2/27) (Table 5, Additional file 1: Table S3 and Additional file 2: Fig. S1). After ablation, recurrent thrombus was not detected in patients who discontinued oral anticoagulants and maintained sinus rhythm. Thrombi did not develop in patients with recurrent atrial tachyarrhythmia who maintained sustainable anticoagulant or antiplatelet drug therapy. As for LAAFV, it presented with a significant increase among patients with sustainable sinus rhythm (n = 15, pre and post-CA difference 17.46 ± 14.81 cm/s, *p* < 0.001), no significant change was observed in recurrent tachyarrhythmia patients (n = 12, 4.46 ± 10.84 cm/s, *p* > 0.05) (Table 5, Fig. 3 and Additional file 1: Table S3). Similarly, LAD presented with a significant decrease among patients freedom from any atrial arrhythmia (n = 15, pre and post-CA difference 1.47 ± 2.59 mm, *p* = 0.045), no significant change was observed in recurrent tachyarrhythmia patients (n = 12, 1.33 ± 2.23 mm, *p* > 0.05) (Table 5 and Additional file 1: Table S3). The exact mean value of the LAAFV in sinus rhythm and AF rhythm prior ablation showed significant difference (sinus and AF, 47.78 ± 15.03 cm/s vs. 30.94 ± 12.14 cm/s, *p* = 0.005), there was no significant difference in LAAFV between sinus rhythm and AF rhythm post ablation (sinus and AF, 48.28 ± 20.62 cm/s vs. 39.20 ± 15.98 cm/s, *p* > 0.05).

**Table 5:** TEE results prior and post-CA in 27 patients

Age (years)*	Diagnosis	LAAFV prior/post CA (cm/s)	LVEF prior/post CA (%)
68	PerAF	22.5/23.3	38/42
63	PerAF	16.7/26.8	45/47
69	PerAF	15.6/27.9	35/52
69	PerAF	45.2/57.1	47/45
66	PerAF	19.4/41.1	58/55
45	PaAF	50.3/ 69.6	57/60
68	PaAF	72.3/ 74.2	64/58
52	PerAF	42.1/50.5	55/60
48	PerAF	25.2/30.1	40/47
51	PerAF	41.6/ 53.3	30/43
57	PaAF	49.0/59.0	60/55
59	PerAF	27.5/ 45.6	60/63
65	PerAF	19.4/25.9	60/65
62	PerAF	58.6/36.0	52/55
52	PerAF	23.9/23.6	48/50
64	PerAF	31.6/32.9	76/75
54	PerAF	42.7/43.1	65/61
73	PerAF	46.1/58.6	70/65
55	PerAF	24.9/50.6	60/55
39	PerAF	36.8/55.4	60/63
51	PaAF	32.8/52	60/65
76	PaAF	52.2/48	42/40
43	PaAF	61.6/116.8	58/60
52	PaAF	27.6/48.6	60/65
53	PaAF	36.4/40.6	76/70
58	PerAF	19.4/25.9	60/60
54	PerAF	28.6/68.9	50/55

*TEE* transesophageal echocardiography, *LAAFV* left atrial appendage flow velocity, *LVEF* LV eject fraction, *PerAF* persistent AF, *PaAF* paroxysmal AF

*Prior CA

## Discussion

This study addressed the risk factors of atrial thrombus in patients, confirming that the presence of intracavitary thrombus, unlike the CHA_2_DS_2_-VASc score, is independently associated with the type of AF. During follow-up, the recurrence rate of atrial tachyarrhythmia in patients with atrial thrombus was higher after CA. For patients with a history of cardiac thrombus, CA improved the LAAFV, particularly in those with sustainable sinus rhythm.

### The risk factors of thrombus formation

In recent years, numerous pieces of literature have literature has examined the risk factors of intracardiac thrombus and the value of CHA_2_DS_2_-VASc scores in AF patients, and have yielded varying results. Multiple studies have shown that the risk of thromboembolism in patients with non-paroxysmal AF is much higher than that in patients with paroxysmal AF [17–19]. A retrospective study [20] of 2695 NVAF patients in China suggested that both the CHADS_2_ and CHA_2_DS_2_-VASc scores was limited in predicting left atrial appendage thrombus (LAAT) in patients with NVAF. The researchers developed a new composite model including previous stroke/TIA, non-paroxysmal AF, moderate to severe left ventricular systolic dysfunction, left atrial enlargement, and cardiomyopathy, improving the value of the AUC when compared to the CHA_2_DS_2_-VASc scoring system. Kaplon et al.’s retrospective study [21] reported that LAAT formation is associated with AF type and renal dysfunction, besides the variables of the CHA_2_DS_2_-Vasc score. They proposed CHA_2_DS_2_-VASc-RAF scores in which non-paroxysmal AF were heavily represented in the scoring system and were thought to have a significant impact on atrial thrombosis. This study suggests that patients with non-paroxysmal AF who have a CHA_2_DS_2_-VASc scores of 0 or 1 should not be classified as low-risk for thromboembolism and should also require anticoagulant therapy. Beata et al. [22] inferred that non-paroxysmal AF and eGFR < 60 mL/min/1.72 m^2^ are primary predictors of LAAT in AF patients with lower CHA_2_DS_2_-VASc score, which is partly consistent with our study. The effect of eGFR on LAAT may be due to the close association of kidney disease with other variables of CHA_2_DS_2_-VASc scores, such as heart failure, hypertension, and diabetes mellitus. In a clinical study [23] that addressed the association between oral anticoagulant (OAC) therapy and intracardiac thrombi focused primarily on the role of OACs as an independent determinant of intracardiac thrombus formation. Yet, even optimal OACs could not prevent thrombi formation entirely. A recent study [24] reported that among patients with a CHA_2_DS_2_-VASc score > 1, the prevalence of thrombus or sludge in LAA was independent of the CHA_2_DS_2_-VASc score value. The slower LAAFV, presence of SEC, longer duration of arrhythmia and some other factors can increase the risk for the presence of thrombus in LAA. Therefore, further studies are needed to explore more risk factors affecting atrial thrombosis, the timing and necessity of OAC therapy require further validation with a combination of various conditions clinically, rather than simply relying on CHA_2_DS_2_-VASc scores.

Additionally, researchers found additional independent predictors for the occurrence of intracardiac thrombus, including a history of embolism, hypertension, BMI, reduced atrial appendage flow, and the morphology of LAA [25–28]. Some of the aforementioned findings were not obtained in our study, which may be related to the younger-aged patients and failure to account for the duration, severity, or treatment status of underlying diseases, i.e., congestive heart failure, hypertension, and diabetes mellitus. Most studies adhere to pre-ablation anticoagulation, our institution does not, thus the singular effect of CA on thrombus formation is more clearly examined. Additional parameters that can affect atrial thrombus formation were not included in our analysis since we were unable to retrieve the relevant data. Although some risk factors cannot be optimized, they are helpful for the early identification of high-risk groups.

Because it is easy to form thrombus in LAA in patients with AF, especially small mural thrombus, according to the guidelines, such as ESC guidelines [11], therapeutic OAC for at least 3 weeks or the use of TEE to exclude LA thrombus before ablation should be considered to prevent thromboembolism. In our center, TEE was routinely performed within 24 h prior to CA, 3-week pre-ablation anticoagulation was omitted. There is statistical difference in prior anticoagulation in patients with or without atrial thrombus (0% vs. 9%, *p* = 0.046). However, we were unable to perform further multivariate regression analyses due to 0% prior anticoagulation rate in the thrombosis group, so the effect of prior anticoagulation on the thrombus formation cannot be completely excluded, which is also a limitation in the statistics of our study.

According to Virchow’s triad, most patients with AF may have the three risk factors of thrombus formation [29]. Since persistent AF can also lead to slower LAAFV, atrial enlargement, and atrial fibrosis, therefore, screening for intracardiac thrombus should be prioritized in order to formulate reasonable individualized prevention and treatment strategies.

### Efficiency of CA in rhythm control after the thrombus has resolved

In patients with AF, CA is more effective in rhythm control than antiarrhythmic drugs [8]. The remodeling of the left atrium may mainly influence the efficacy of CA [30]. Nishizaki and colleagues believed that AF recurrence-free rate of CA in patients with a history of cardioembolic stroke showed no significant difference compared with those without cardioembolic stroke [31]. In the current study, the success rate of CA (35/58, 60.34%) in patients with a history of LAAT was lower than the no-thrombus group (1258/1668, 75.42%), which may be associated with the high LA diameter and the incidence of persistent AF.

### Long-term effect of CA on thrombus formation after the thrombus has resolved

AF predisposes patients to thrombogenic tendency; this mechanism includes: abnormal flow, endocardial endothelium structural changes, and blood constitutes, i.e., Virchow’ triad of thrombus formation [10, 29]. As for AF patients, abnormal changes in intra-atrial flow are noticeable by stasis (i.e., low velocity of flow) in the left atrium and presented as SEC, which is highly associated with the thrombus formation [32]. Machino-Ohtsuka and colleagues found that successful CA improved the LAA function, left atrial volume decreased by ≥ 15% during follow-up [33]. Kusa et al. reported that long-term maintenance of sinus rhythm for persistent AF did not assure the recovery of LAAFV in patients with a high CHA_2_DS_2_-VASc score [9]. Our study investigated the long-term effect of CA on LAAFV and thrombus formation in patients with a previous history of thrombus. Although our sample size was small, we found that the LAAFV improved in patients who maintained a sustainable sinus rhythm. It is worth noting that the CHA_2_DS_2_-VASc score was low in a large majority of our patient population (16/27) because patients with many complications are usually not referred for CA. Low LAAFV is an independent predictor of stroke, as reported by Lee et al. [34]. Therefore, the improvement of LAAFV in the present study may be associated with preventing clinical ischemic stroke events and silent stroke lesions in those patients with a history of thrombus. The high rate of white matter hyperintensity lesions observed in our study (92.6%) is possibly due to age [35], AF [36], or the interventional procedure [16]. A large multicenter study by David Conen et al. reported a 99% lesion rate in the AF patient population [36], with no clinical significance to cognitive function. These results may imply that successful rhythm control therapy improves thrombogenic factors, namely LAAFV and SEC. Thus, no further stroke preventive measures, such as anticoagulants or LAA occlusion are required, especially in patients with a low CHA_2_DS_2_-VASc score. The patients who agreed to the follow-up TEE examination had relatively low CHA_2_DS_2_-VASc scores. It is a preliminary finding that a successful CA improved the substrate of thrombogenesis.

### Clinical implications

Thromboembolic events affect the prognosis of patients with AF. The CHA_2_DS_2_-VASc score is the most widely used risk stratification scheme clinically. However, the relationship between the CHA_2_DS_2_-VASc score and LAAT is yet to be determined. A study showed that the incidence of atrial thrombosis diagnosed by TEE was about 2% even after patients received adequate anticoagulant therapy [37]. Most AF patients in China experience insufficient cognition of anticoagulant therapy and have low tolerance to anticoagulant drugs and high bleeding tendency. As a result, some patients are influenced by poor compliance and fail to achieve reasonable dosage in clinical practice [12]. Therefore, it is necessary to establish a reliable method to accurately identify the high-risk population of atrial thrombosis, so as to provide optimal anticoagulant therapy for patients.

CA is an effective modality to restore sinus rhythm in AF. Several non-randomized studies, including ours, have found that the incidence of embolism in patients with sinus rhythm after CA is lower than patients experiencing recurrence [15, 38]. The evidence reported in the present study showed that CA increased the LAAFV and improved the thrombus formation matrix, which may be one of the mechanisms that reduced embolic events. Regarding the patient who maintained sinus rhythm after CA, LAAFV increased notably. Therefore, the effect of successful CA on the thrombotic matrix requires further investigation. Investigation into the independent risk factors of thrombus formation and bleeding is essential to identify high-risk groups.

### Limitations

This study has the following limitations: Firstly, this is a single-center, case–control study, which is prone to confounding factors. The influencing factors not included in the study may be potential risk factors for cardiac thrombus. Secondly, our analysis did not include the duration, severity, and treatment regimens of underlying diseases. Thirdly, the number of participants with thrombus that underwent repeat TTE are low, making the conclusions of this paper limited.

## Conclusions

Our results support that persistent AF is an independent risk factor for thrombus formation. Successful CA may improve the LAAFV and thereby decrease the risk of intracardiac thrombus formation. Additional multicenter prospective studies are required to identify further management measures to prevent recurrent thrombus formation.

## Supplementary Information

**Additional file 1: Table S1.** Baseline clinical and demographic characteristics and thrombus resolution management. **Table S2.** Catheter ablation procedure and outcomes. **Table S3.** TEE and brain MRI results, post-CA in 27 patients**.**

**Additional file 2: Fig. S1.** Brain MRI findings in the same patient presenting with lesions—white matter hyperintensity (white arrow) showing an increased signal in T2 sequence (**A**), decreased signal in T1 sequence (**B**), increased signal in FLAIR (**C**) and iso-intense signal in DWI (**D**).

## Abbreviations

AF: Atrial fibrillation
CA: Catheter ablation
LAAFV: Left atrial appendage flow velocity
TEE: Transesophageal echocardiography
VKA: Vitamin K antagonists
DOAC: Direct-acting oral anticoagulants
LA: Left atrium
LAA: Left atrial appendage
LAD: Left atrial dimension
LAAT: Left atrial appendage thrombus
SEC: Spontaneous echo contrast
eGFR: Estimated glomerular filtration rate
