# Supplementary material for: The risk factors of thrombus formation and the effect of catheter ablation on repetitive thrombus formation in patients with atrial fibrillation: a single center retrospective study in China
Source: BMC Cardiovasc Disord. 2023 Jan 17;23:28. doi: 10.1186/s12872-023-03050-z (PMC9843887; doi:10.1186/s12872-023-03050-z)
Supplement: Supplementary file 1 — Additional file 1: Table S1. Baseline clinical and demographic characteristics and thrombus resolution management. Table S2. Catheter ablation procedure and outcomes. Table S3. TEE and brain MRI results, post-CA in 27 patients. [file 12872_2023_3050_MOESM1_ESM.pdf]

Supplementary Table 1 Baseline clinical and demographic characteristics and thrombus resolution management

Age (years)	Gender	Diagnosis	BMI	CHA2DS2-VASc	HAS-BLED	Thrombus size (cm^2)/ location	Spontaneous echo contrast	LAAFV (cm/s)	Drugs for resolution	TTR for Warfarin (%)	Duration to resolution (days)	Major bleeding	Embolic event
68	male	PerAF	34.4	2	2	2.31/LAA	Massive in LA	23.6	Dabigatran	-	90	None	None
63	female	PerAF	30.0	1	0	0.78/LAA	Massive in LA	35.6	Dabigatran	-	128	None	None
69	female	PerAF	29.4	4	3	0.84/LAA	Massive in LA	13.2	Warfarin/ Dabigatran	33.3	208	None	None
69	female	PerAF	27.2	3	1	1.53/LAA	Medium in LA	15.3	Dabigatran	-	92	None	None
66	male	PerAF	26.4	3	2	0.70/LAA	None	21.2	Warfarin	65.0	114	None	None
45	male	PaAF	25.9	1	0	3.00/LAA	None	45.4	Dabigatran	-	67	None	None
68	male	PaAF	26.6	5	2	0.48/LAA	None	61.3	Warfarin	70.0	93	None	None
52	female	PerAF	25.0	1	0	1.19/LAA	Mild in LA	18.1	Dabigatran	-	87	None	None
48	male	PerAF	27.5	1	0	0.70/LAA	None	61.9	Dabigatran	-	95	None	None

51	male	PerAF	28.6	3	0	0.98/LAA	None	43.3	Warfarin	90.0	89	None	None
57	male	PaAF	35.1	2	2	0.60/LAA	Massive in LA	12.6	Warfarin/ Dabigatran	50.0	365	None	None
59	male	PerAF	25.8	0	0	1.76/LAA	None	29.8	Warfarin	68.0	236	None	None
65	female	PerAF	28.1	3	2	1.08/LAA	Medium in LA	26.4	Warfarin	65.0	128	None	None
62	male	PerAF	32.2	1	0	0.36/LAA	None	63.1	Warfarin	80.0	95	None	None
52	male	PerAF	29.7	1	1	1.28/LAA	Massive in RA and LA	21.8	Warfarin	60.0	88	None	None
64	female	PerAF	29.3	2	1	1.12/LAA	Mild in LA	27.3	Warfarin	55.0	85	None	None
54	male	PerAF	27.7	1	0	0.54/LAA	None	41.3	Dabigatran	-	97	None	None
73	male	PerAF	27.7	3	1	0.65/RA	None	47.8	Dabigatran	-	96	None	None
55	male	PerAF	24.8	1	1	0.32/LAA	None	27.6	Dabigatran	-	117	None	None
39	male	PerAF	27.8	1	0	0.35/LAA	Mild in LA	29.6	Warfarin	75.0	100	None	None
51	female	PaAF	23.7	2	0	0.45/LAA	Mild in LA	27.3	Warfarin	50.0	72	None	None
76	female	PaAF	27.3	5	1	5.32/RA	Mild in LA	60.0	Dabigatran	-	68	None	None

43	female	PaAF	24.5	1	0	1.28/RA	None	65.3	Dabigatran	-	102	None	None
52	male	PaAF	23.2	2	0	0.42/LAA	Medium in LA	30.9	Dabigatran	-	98	None	None
53	male	PaAF	27.1	1	0	1.76/RA	Mild in RA	37.8	Dabigatran	-	76	None	None
58	male	PerAF	22.5	0	0	0.99/LAA	Mild in LA	17.3	Warfarin	66.7	166	None	None
54	male	PerAF	21	1	0	0.60/LAA	Massive in LA	32.6	Dabigatran	-	98	None	None

BMI-body mass index, LAA-left atrial appendage, LAAFV-LAA flow velocity, TTR-time in therapeutic range, RA-right atrium, PerAF-persistent atrial fibrillation, PaAF-paroxysmal atrial fibrillation

Supplementary Table 2 Catheter ablation procedure and outcomes

Age (years)*	Diagnosis	AF duration since first diagnosis (months)	CA operation	Electrical cardioversion	CA Times	Recurrence type after last CA	Recurrence time after last CA (months)	Duration of follow-up from last CA (months)	Continuous antiarrhythmic drugs	Continuous anticoagulants / antiplatelet drugs	Embolic event	LA diameter prior CA (mm)
68	PerAF	108	CPVI+CFAE	Yes	1	PaAF	4	77	Metoprolol	Aspirin	None	52
63	PerAF	120	CPVI+CFAE	Yes	1	No	-	67	No	No	None	45
69	PerAF	156	CPVI+CFAE +LR+MI	Yes	2	PaAF	12	57	No	Aspirin	None	43
69	PerAF	36	CPVI+CFAE +LR	Yes	1	No	-	56	No	Aspirin	None	40
66	PerAF	36	CPVI+CFAE	No	1	No	-	56	No	No	None	43
45	PaAF	84	CPVI+TI	No	1	No	-	46	No	No	None	40
68	PaAF	36	CPVI+CFAE	Yes	1	No	-	45	No	Aspirin	None	36
52	PerAF	48	CPVI+CFAE	Yes	1	PerAF	3	40	No	No	None	50
48	PerAF	3	CPVI+CFAE +LR+MI	Yes	1	PaAF	22	39	Metoprolol	Dabigatran	None	47

51	PerAF	9	CPVI+LR+MI	Yes	1	No	-	38	Metoprolol	No	None	49
57	PaAF	60	CPVI+LR+MI+TI	Yes	2	PaAF	11	49	Metoprolol	Aspirin	None	50
59	PerAF	60	CPVI+CFAE+LR+MI	Yes	1	No	-	34	No	No	None	55
65	PerAF	6	CPVI+CFAE+LR+MI	Yes	1	No	-	29	No	Aspirin	None	46
62	PerAF	36	CPVI+CFAE	Yes	1	PerAF	12	28	Metoprolol	Warfarin	None	51
52	PerAF	60	CPVI+CFAE+LR+MI	Yes	1	PerAF	3	27	Metoprolol	Rivaroxaban	None	45
64	PerAF	12	CPVI+CFAE+LR+MI	Yes	1	No	-	26	Metoprolol	No	None	46
54	PerAF	48	CPVI+CFAE+LR+MI	Yes	1	No	-	24	Metoprolol	No	None	43
73	PerAF	12	CPVI	No	1	PaAF	3	24	No	Warfarin	None	38
55	PerAF	6	CPVI+LR+MI	Yes	1	No	-	17	No	No	None	41
39	PerAF	120	CPVI+CFAE+LR+MI	Yes	1	No	-	14	Metoprolol	No	None	39
51	PaAF	24	CPVI	No	1	No	-	14	No	No	None	37
76	PaAF	192	CPVI	No	2	PaAF	24	43	No	Aspirin	None	39

43	PaAF	15	CPVI	No	1	No	-	25	Bisoprolol	No	None	37
52	PaAF	96	CPVI	No	1	PaAF	10	24	Metoprolol	No	None	35
53	PaAF	16	CPVI+TI	No	1	PaAF	6	22	Metoprolol	Aspirin	None	33
58	PerAF	1	CPVI+CFAE +LR+MI	Yes	2	PaAF	15	21	Metoprolol	No	None	43
54	PerAF	60	CPVI+CFAE +LR+MI	Yes	1	No	-	20	No	No	None	44

AF-atrial fibrillation, CA-catheter ablation, LA-left atrium, PerAF-persistent AF, PaAF-paroxysmal AF, CPVI-circumferential pulmonary vein isolation, CFAE- complex fractionated atrial electrogram, LR-LA roof, MI-mitral isthmus, TI-tricuspid isthmus

* prior RFCA

Supplementary Table 3 TEE and brain MRI results, post-CA in 27 patients

Age (years)*	Diagnosis	Recurrence time after last CA (months)	Thrombus formation post CA	Spontaneous echo contrast post CA	Rhythm when TEE performed prior/post CA	LAAFV prior /post CA (cm/s)	LA diameter prior/ post CA (mm)	LVEF prior/ post CA (%)	Brain MRI findings
68	PerAF	4	No	No	AF/Sinus	22.5/23.3	45/45	38/42	white matter hyperintensity
63	PerAF	-	No	No	AF/Sinus	16.7/26.8	42/39	45/47	white matter hyperintensity
69	PerAF	12	No	No	AF/AF	15.6/27.9	43/44	35/52	white matter hyperintensity
69	PerAF	-	No	No	AF/Sinus	45.2/57.1	37/35	47/45	white matter hyperintensity
66	PerAF	-	No	No	AF/Sinus	19.4/ 41.1	43/41	58/55	softening lesions
45	PaAF	-	No	No	Sinus/Sinus	50.3/ 69.6	40/40	57/60	Normal

68	PaAF	-	No	Mild in LA	Sinus/Sinus	72.3/ 74.2	36/37	64/58	white matter hyperintensity
52	PerAF	3	No	No	AF/AF	42.1/50.5	35/40	55/60	softening lesions
48	PerAF	22	No	No	AF/Sinus	25.2/30.1	43/41	40/47	white matter hyperintensity
51	PerAF	-	No	No	AF/Sinus	41.6/ 53.3	49/47	30/43	white matter hyperintensity
57	PaAF	11	No	No	Sinus/Sinus	49.0/59.0	35/38	60/55	Normal
59	PerAF	-	No	No	AF/Sinus	27.5/ 45.6	55/50	60/63	softening lesions
65	PerAF	-	No	No	AF/Sinus	19.4/25.9	47/46	60/65	white matter hyperintensity
62	PerAF	12	No	No	AF/Sinus	58.6/36.0	51/52	52/55	white matter hyperintensity
52	PerAF	3	No	No	AF/Sinus	23.9/23.6	45/48	48/50	white matter hyperintensity
64	PerAF	-	No	No	AF/Sinus	31.6/32.9	33/35	76/75	white matter hyperintensity
54	PerAF	-	No	No	AF/Sinus	42.7/43.1	43/39	65/61	white matter hyperintensity
73	PerAF	3	No	No	AF/Sinus	46.1/58.6	38/40	70/65	white matter hyperintensity
55	PerAF	-	No	No	AF/Sinus	24.9/50.6	41/40	60/55	white matter hyperintensity

39	PerAF	-	No	No	AF/Sinus	36.8/55.4	39/38	60/63	white matter hyperintensity
51	PaAF	-	No	No	Sinus/Sinus	32.8/52	35/33	60/65	white matter hyperintensity
76	PaAF	24	No	No	Sinus/Sinus	52.2/48	45/48	42/40	softening lesions
43	PaAF	-	No	No	Sinus/Sinus	61.6/ 116.8	37/41	58/60	white matter hyperintensity
52	PaAF	10	No	No	Sinus/Sinus	27.6/48.6	35/36	60/65	white matter hyperintensity
53	PaAF	6	No	Medium in RA	Sinus/Sinus	36.4/40.6	33/35	76/70	softening lesions
58	PerAF	15	No	Mild in LA	AF/Sinus	19.4/25.9	43/40	60/60	white matter hyperintensity
54	PerAF	-	No	Medium in LA	AF/Sinus	28.6/68.9	44/38	50/55	white matter hyperintensity

TEE-Transesophageal echocardiography, MRI-magnetic resonance imaging, RFCA-radiofrequency catheter ablation, LAAFV-left atrial appendage flow velocity, LA-left atrium, LV-left ventricle, LVEF-LV eject fraction, PerAF-persistent AF, PaAF-paroxysmal AF, MRI-magnetic resonance imaging

* prior RFCA
