# Supplementary figures and images for: The risk factors of thrombus formation and the effect of catheter ablation on repetitive thrombus formation in patients with atrial fibrillation: a single center retrospective study in China
Source: BMC Cardiovasc Disord. 2023 Jan 17;23:28. doi: 10.1186/s12872-023-03050-z (PMC9843887; doi:10.1186/s12872-023-03050-z)

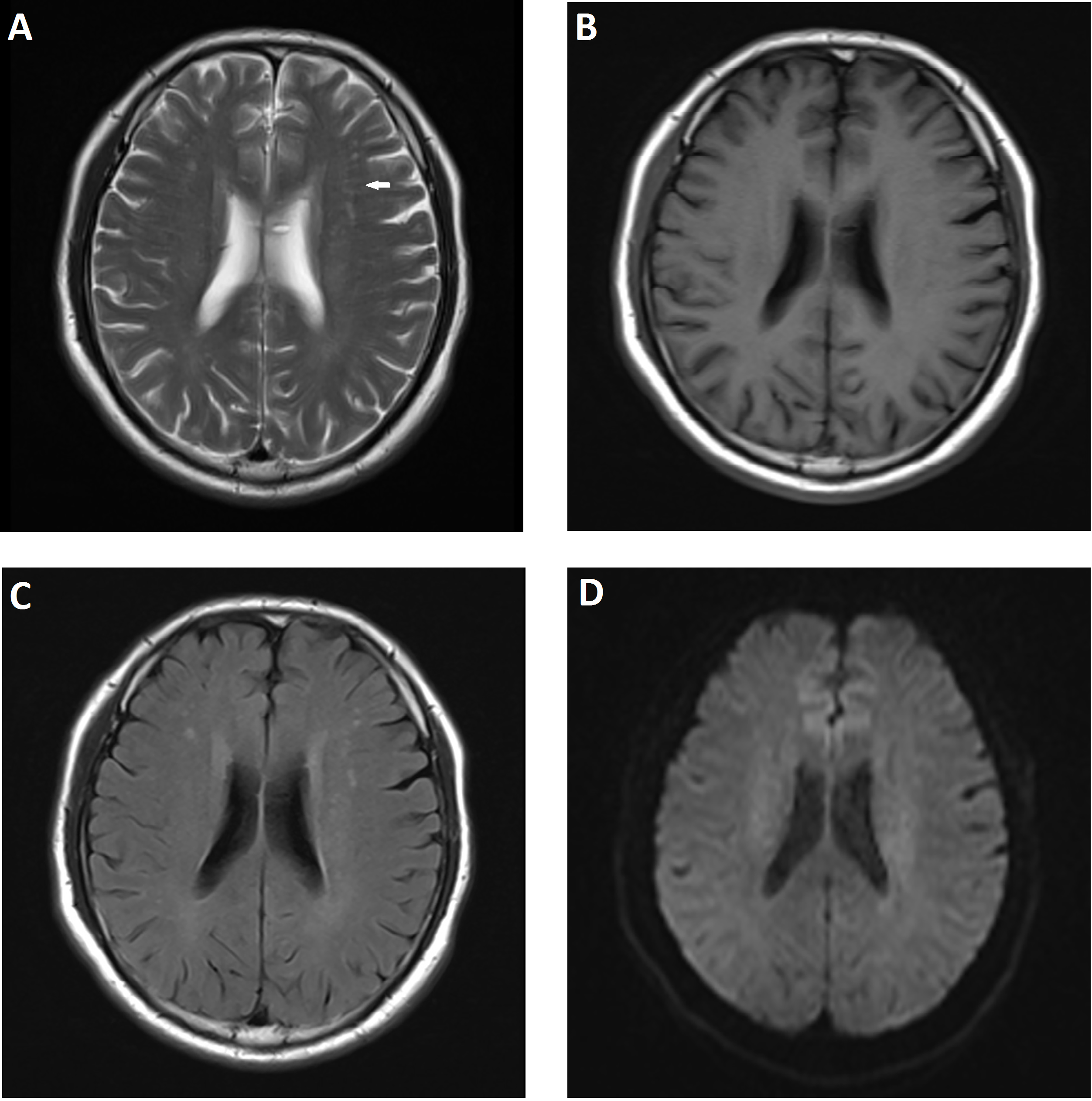

Supplement: Supplementary file 2 — Additional file 2: Fig. S1. Brain MRI findings in the same patient presenting with lesions—white matter hyperintensity (white arrow) showing an increased signal in T2 sequence (A), decreased signal in T1 sequence (B), increased signal in FLAIR (C) and iso-intense signal in DWI (D). [file 12872_2023_3050_MOESM2_ESM.tif]
